# Survival of Neoadjuvant and Adjuvant Therapy Compared With Surgery Alone for Resectable Esophageal Squamous Cell Carcinoma: A Systemic Review and Network Meta-Analysis

**DOI:** 10.3389/fonc.2021.728185

**Published:** 2021-10-20

**Authors:** Zeliang Ma, Meng Yuan, Yongxing Bao, Yang Wang, Yu Men, Zhouguang Hui

**Affiliations:** ^1^ Department of Radiation Oncology, National Cancer Center/National Clinical Research Center for Cancer/Cancer Hospital, Chinese Academy of Medical Sciences and Peking Union Medical College, Beijing, China; ^2^ Department of VIP Medical Services, National Cancer Center/National Clinical Research Center for Cancer/Cancer Hospital, Chinese Academy of Medical Sciences and Peking Union Medical College, Beijing, China

**Keywords:** esophageal squamous cell carcinoma, treatment, systemic review, network meta-analysis, survival

## Abstract

**Objective:**

The optimal treatment for resectable esophageal squamous cell carcinoma (ESCC) remains controversial. Surgery is the primary treatment but with poor results. Attempts to improve patient survival have been made by introducing chemotherapy, radiotherapy, or both. However, randomized comparisons for all these strategies are not always available. This network meta-analysis compared the overall survival of neoadjuvant and adjuvant therapy with surgery alone to identify the most effective approach.

**Methods:**

We systematically searched electronic databases (PubMed, Embase, and Cochrane Library) for relevant studies published before April 2021. Only phase II and III randomized controlled trials comparing the following treatments were included: surgery alone, neoadjuvant chemotherapy (NCT), radiotherapy (NRT) or chemoradiotherapy (NCRT), adjuvant chemotherapy (ACT), radiotherapy (ART), or chemoradiotherapy (ACRT). The hazard ratios (HR) and 95% confidence intervals (CIs) of overall survival (OS) was identified as the measurement of effectiveness. A network meta-analysis was conducted to synthesize the evidence under the Bayesian framework, and the relative effects of all possible comparisons were made. The ranking analysis was performed to support the decision in clinical practice.

**Results:**

A total of 19 relevant trials with 3,749 patients were identified. Compared with surgery alone, NCRT (HR 0.76, 95% CI 0.65–0.89) and NCT (HR 0.81, 95% CI 0.70–0.94) significantly improved OS, while other treatments, including NRT (HR 0.86, 95% CI 0.66–1.08), ACRT (HR 0.73, 95% CI 0.49–1.08), ACT (HR 0.96, 95% CI 0.75–1.21), and ART (HR 0.86, 95% CI 0.66–1.14), provided no significant survival advantage. None of the neoadjuvant and adjuvant treatments showed a statistically significant difference in OS to each other when compared in pairs.

**Conclusion:**

For resectable esophageal squamous cell carcinoma, this network meta-analysis showed that NCRT may be the optimal strategy, NCT may be the second choice, while other multimodality treatments could not improve OS compared with surgery alone. It remains unclear whether ESCC will benefit from adding radiotherapy into the neoadjuvant treatment.

**Systematic Review Registration:**

We registered this meta-analysis protocol at the prospective register of systematic reviews, PROSPERO https://www.crd.york.ac.uk/prospero/display_record.php?RecordID=172745 (Identification code: CRD42020172745).

## Introduction

Esophageal cancer is the sixth most common cause of cancer-related death worldwide, and esophageal squamous cell carcinoma (ESCC) is the most common histology subtype (1). Surgery is the mainstream treatment for resectable ESCC; however, the overall survival remains unsatisfactory if treated with surgery alone (2). Multimodality treatments based on surgery have been investigated to improve the overall survival (OS), including neoadjuvant or adjuvant chemotherapy, radiotherapy, or chemoradiotherapy. However, randomized comparisons for all these strategies are not always available. The optimal treatment for resectable ESCC remains controversial.

Network meta-analysis could simultaneously compare the overall survival of different treatments through direct and indirect comparisons to identify the most effective approach. Several network meta-analyses were conducted but with inconsistent results and some major drawbacks (3–5), resulting in insufficient evidence. Besides, several essential trials have been published in recent years (6–9), and therefore, it is necessary to perform a rigorous and updated review. This network meta-analysis compared overall survival after multimodality treatments or surgery alone to identify the most effective approach.

## Methods

### Systematic Review

#### Searching Literature

We carried out a systematic search of available literature and reported results in line with PRISMA (Preferred Reporting Items for Systematic Reviews and Meta-Analyses) (10) and AMSTAR (Assessing the methodological quality of systematic reviews) (11) Guidelines. We registered this meta-analysis protocol at the prospective register of systematic reviews, PROSPERO (Identification code: CRD42020172745). According to the pre-specified searching strategy and inclusion criteria, we systematically searched electronic databases (PubMed, Embase, and Cochrane Library) for relevant studies published before April 2021. The searching strategy was provided in **Supplement 1**.

#### Inclusion Criteria

1) Phase II/III RCTs with a sample size larger than 50 patients;
2) Trials that enrolled patients with resectable thoracic ESCC;
3) Trials that compared different treatments [surgery alone, neoadjuvant chemotherapy (NCT), radiotherapy (NRT) or chemoradiotherapy (NCRT), adjuvant chemotherapy (ACT), radiotherapy (ART) or chemoradiotherapy (ACRT)];
4) Total radiation dose should be more than 30 Gy;
5) Trials that designed OS as the primary endpoint;
6) Only full-text articles were included; if multiple publications of the same trial were retrieved, the most recent and informative paper was included.

#### Quality Assessment

Two reviewers independently assessed the quality of all trials using a revised Cochrane risk of bias tool for randomized trials (12), while another reviewer served as a referee in case of controversies. The full scale is constituted by the following domains: randomization process, deviations from intended interventions, missing outcome data, measurement of the outcome, selection of the reported result, and overall bias. According to the detailed guidance of RoB 2, each domain could be judged as any of the three levels: low risk, high risk, or unclear risk of bias.

The funnel plot was used to evaluate potential publication bias (13).

#### Data Extraction

Two reviewers extracted the data independently. Any discrepancies were discussed with a third reviewer and reached a consensus ultimately. The data obtained from each article are listed as follows: (1) first author**’**s name, (2) publication year, (3) sample size, (4) tumor location, (5) treatment regimens, and (6) survival data.

### Statistical Analysis

Overall survival (OS) was identified as the measurement of effectiveness and expressed as hazard ratios (HR), which considered the timing and censoring of survival status. To obtain the HR and its standard error, three approaches were applied: (1) For studies that reported the summary statistics, HR was directly collected, and standard error was calculated from 95% confidence interval (CI). (2) In the absence of summary statistics, some studies published the Kaplan–Meier curve with at-risk table. Using Tierney’s method, survival rate and number at risk were extracted from the plot based on the time intervals divided schematically. The number of survival patients, deaths, and censors were estimated for every time interval. HR and standard error were calculated by combining all time intervals. (3) For studies that published the Kaplan–Meier curve with the follow-up information instead of at-risk table, similar steps were applied, whereas the estimate of censor is approximated based on a linear pattern.

Bayesian network meta-analysis was carried out to synthesize all therapeutic options within a mixed treatment comparison framework using R 3.6.0. The random-effects model was prioritized to address the study-specific effects, which were components of the overarching distribution. Uninformative prior distribution was given to all parameters. The node-split method was used to assess the inconsistency. The estimates of relative effects and 95% CI were reported. Besides, the surface under the cumulative ranking scores were calculated as well.

## Results

### Description of Studies Included and Quality Assessment

**Figure 1:**
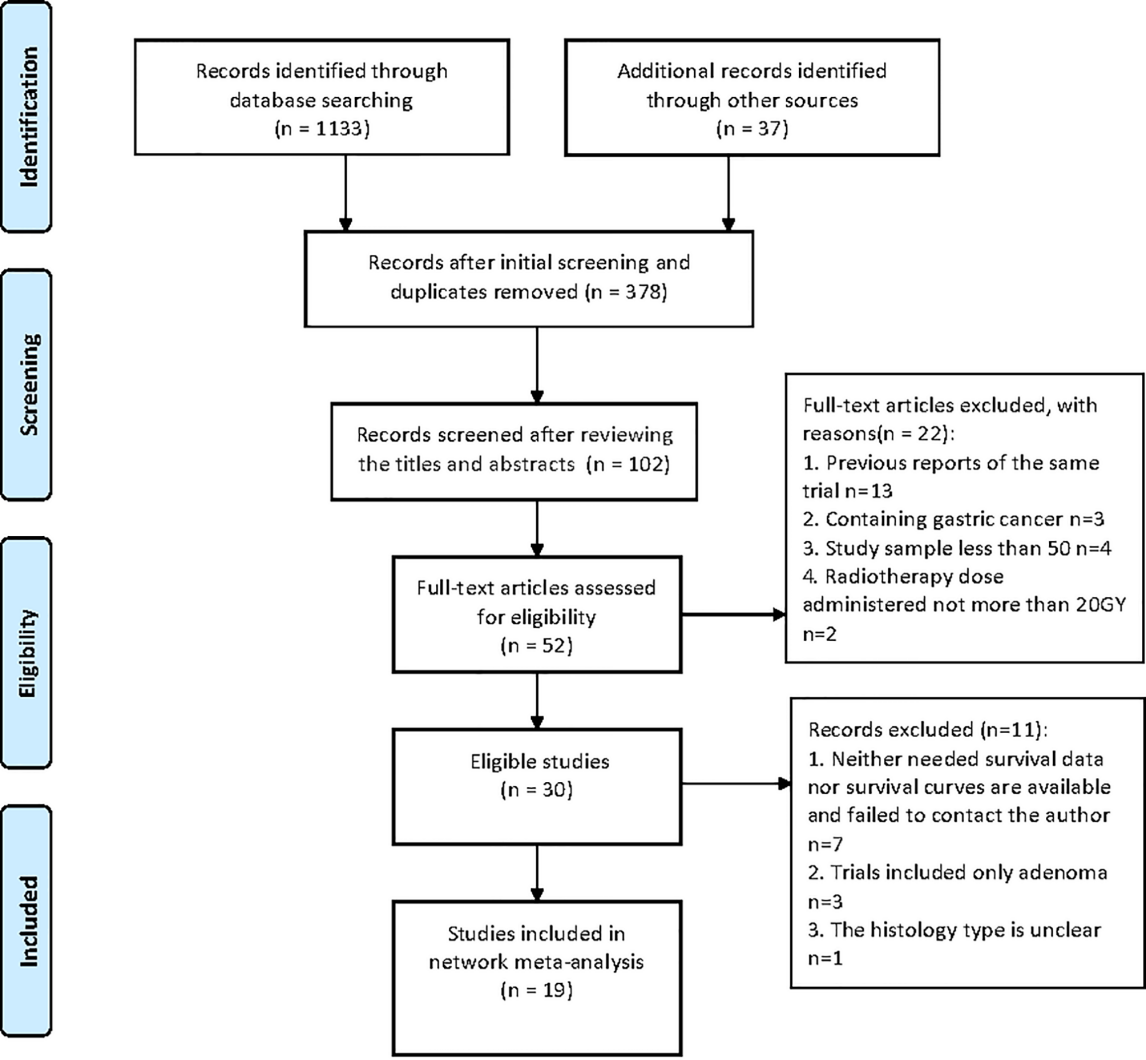
Literature search/PRISMA flow chart.

**Figure 2:**
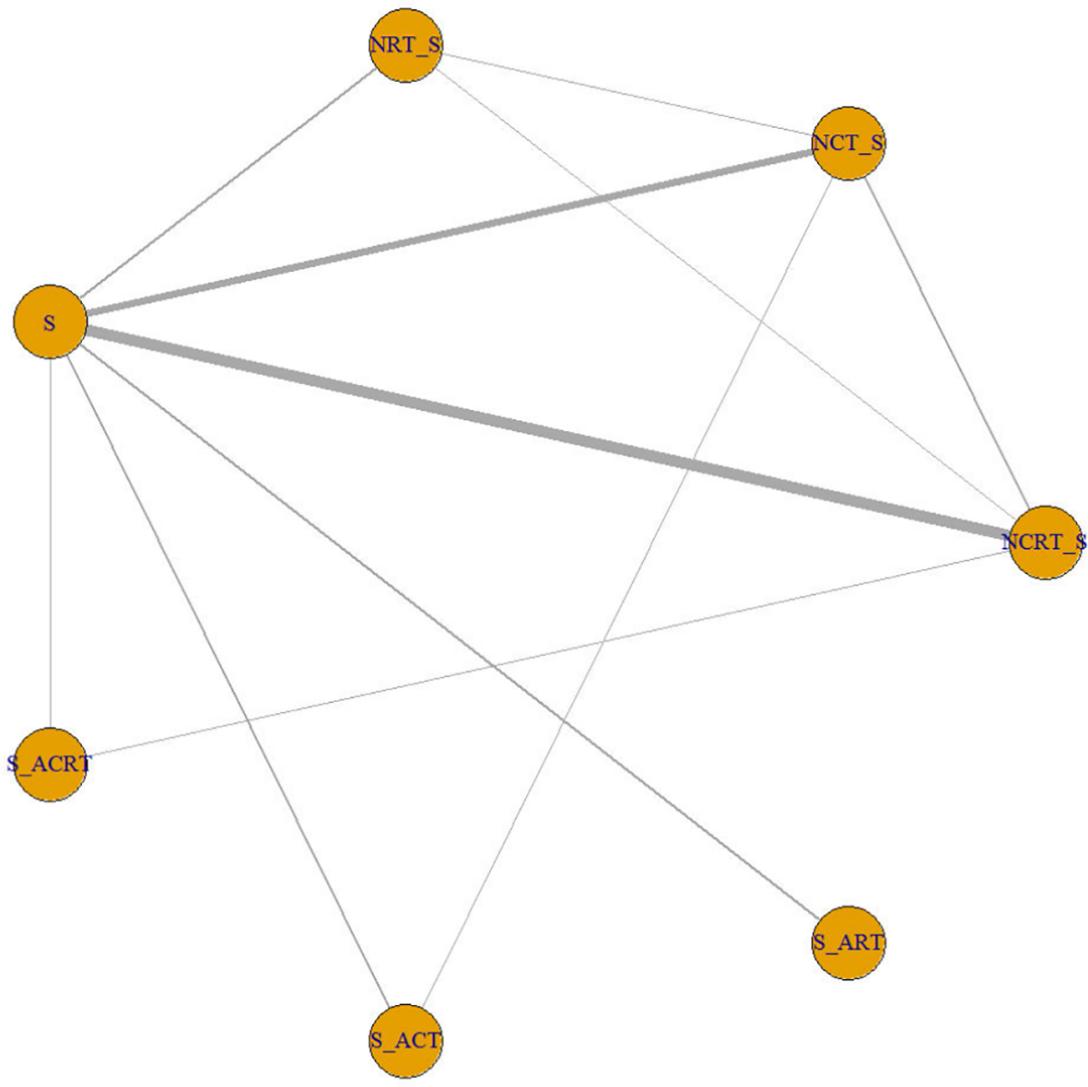
Network plot showing the following different treatment modalities for patients with esophageal squamous carcinoma. The node size is proportional to the total number of patients receiving specific treatment. Each line represents a type of head-to-head comparison. The width of lines is proportional to the number of trials comparing the connected treatments. S, surgery; NCT_S, neoadjuvant chemotherapy followed by surgery; NRT_S, neoadjuvant radiotherapy followed by surgery; NCRT_S, neoadjuvant chemoradiotherapy followed by surgery; S_ACT, surgery followed by adjuvant chemotherapy; S_ART, surgery followed by adjuvant radiotherapy; S_ACRT, surgery followed by adjuvant chemoradiotherapy.

The search and selection process was displayed in a PRISMA diagram (**Figure 1**). Nineteen eligible trials with 3,749 patients were included, and 21 comparisons were generated (Network plot, **Figure 2**). The main features of the studies are shown in **Table 1**.

**Table 1:** Essential characteristics of the studies included in the meta-analysis.

Author	Year of publication	Design	No. pts	Tumor type	No. pts with SCC	RT Schedule	CT Schedule
Allum et al. (14)	2009	NCT-S vs. S	802	AC, SCC, undifferentiated carcinoma	247	NA	Two cycles of Cisplatin 80 mg/m^2^ on day 1 and fluorouracil 1,000 mg/m^2^ daily as a continuous infusion over 96 h repeated every 3 weeks.
Ando et al. (15)	1997	S-ACT vs. S	205	SCC	205	NA	Two cycles of Cisplatin (70 mg/m^2^) and vindesine (3 mg/m^2^) on days 1 and 21.
Ando et al. (16)	2011	S-ACT vs. NCT-S	330	SCC	330	NA	Two cycles of cisplatin 80 mg/m^2^ on day 1; 5-fluorouracil 800 mg/m^2^ on days 1 through 5.
Ando et al. (17)	2003	S-ACT vs. S	242	SCC	242	NA	Two courses of cisplatin (80 mg/m^2^ day1) and fluorouracil (800 mg/m^2^ days 1–5)
Ancona et al. (18)	2001	NCT+S vs. S	94	SCC	94	NA	Two cycles of cisplatin (100 mg/m^2^) and 5-fluorouracil (1,000 mg/m^2^). Cisplatin day 1 and 21. 5-fluorouracil days 1–5 and 21–26.
Bonstra et al. (19)	2011	NCT-S vs. S	169	SCC	169	NA	Two to four cycles of cisplatin, at a dose of 80 mg on day 1, Etoposide, at a dose of 100 mg/m^2^ on day 1 and day 2, followed by etoposide 200 mg/m^2^ orally on days 3 and 5, repeated every 3 weeks.
Bosset et al. (20)	1997	NCRT-S vs. S	282	SCC	282	37 Gy/3.7 Gy/10 f	Two cycles of Cisplatin, at a dose of 80 mg/m^2^, d1–3
Burmeister et al. (21)	2005	NCRT-S vs. S	256	SCC, AC	95	35 Gy/2.3 Gy/15 f	One cycle of 80 mg/m^2^ cisplatin on day 1, 800 mg/m^2^ fluorouracil on days 1–4
Gignoux et al. (22)	1987	NRT-S vs. S	208	SCC	208	33 Gy/3.3 Gy/10 f	NA
Law et al. (23)	1997	NCT-S vs. S	147	SCC	147	NA	Two cycles cisplatin 100 mg/m^2^ d1 + fluorouracil 500 mg/m^2^/day d1–5
Lee et al. (24)	2004	NCRT+S vs. S	101	SCC	101	45.6 Gy/1.2 Gy/38 f	Two cycles of cisplatin (60 mg/m^2^) on days 1 and 21 and 5-FU (1000 mg/m^2^) from days 2–5
Lv et al. (25)	2010	NCRT+S vs. S+ACRT vs. S	238	SCC	238	40 Gy/2 Gy/20 f	Two cycles of PTX (135 mg/m^2^ per day) and DDP (20 mg/m^2^ per day) on days 1–3 and 22–24 of radiotherapy.
Marittte et al. (26)	2014	NCRT+S vs. S	195	AC, SCC	57	45 Gy/1.8 Gy/25 f	Two cycles of fluorouracil (FU) + cisplatin from days 1 to 5 and 29 to 33
Nygaard et al. (27)	1992	S vs. NCT+S vs. NRT+S vs. NCRT+S	186	SCC	186	35 Gy/1.75 Gy/20 f	Two cycles of cisplatin (20 mg/m^2^) and bleomycin (5 mg/m2). Cisplatin days 1–5. Bleomycin day 1
Shapiro et al. (8)	2015	NCRT-S	368	AC, SCC, undifferentiated carcinoma	84	41.4 Gy/1.8 Gy/23 f	Five cycles carboplatin AUC 2 mg/ml/min d1, 8, 15, 22, 29 + paclitaxel 50 mg/m^2^ d1, 8, 15, 22, 29
von Dobeln et al. (28)	2018	NCRT-S vs. NCT-S	181	AC, SCC	50	40 Gy/2 Gy/20 f	Three cycles cisplatin 100 mg/m^2^ d1 + fluorouracil 750 mg/m2/day, d1–5
Xiao et al. (29)	2003	S-ART vs. S	495	SCC	495	60 Gy/30 f, transpositional stomach:50 Gy/25 f	NA
Yang et al. (6)	2018	NCRT-S vs. S	451	SCC	451	40 Gy/2 Gy/20 f	Two cycles vinorelbine 25mg/m2 d1,8 + cisplatin 75mg/m2 d1 q3wks
Zieren et al. (30)	1995	S-ART vs. S	68	SCC	68	55.8 Gy/1.8 Gy/31 f	NA

NA, Not applicable.

We did not find an obvious risk of bias (**Supplement 2**). All included trials were generally comparable in terms of clinical features. We did not find a violation of the transitivity assumption. Inconsistency between direct and indirect evidence was investigated with node-split models. The network design could identify seven closed loops, and a different model was built for each of them. We did not detect a significant difference between direct and indirect treatment, except for the comparison between NRT and NCRT (*p* > 0.05 for all models, **Supplement 3**). We made a funnel plot and found no publication bias (**Supplement 4**).

### Network Meta-Analysis of the Overall Results

The results are shown in **Table 2**. Compared with surgery alone, NCRT (HR 0.76, 95% credible interval 0.65–0.89) and NCT (HR 0.81, 0.70–0.94) showed favorable OS with significant difference, while NRT (HR 0.86, 0.66–1.08), ACRT (HR 0.73, 0.49–1.08), ACT (HR 0.96, 0.75–1.21), and ART (HR 0.86, 0.66–1.14) provided no significant survival advantage. Besides, multimodality treatment did not show a statistically significant difference from each other.

**Table 2:** The treatment effect for all the evaluated treatment options.

	NCRT_S	NCT_S	NRT_S	S	S_ACRT	S_ACT	S_ART
**NCRT_S**	1	1.07 (0.87, 1.32)	1.13 (0.84, 1.48)	1.32 (1.12, 1.54)	0.96 (0.64, 1.44)	1.26 (0.94, 1.67)	1.14 (0.83, 1.57)
**NCT_S**	0.93 (0.76, 1.15)	1	1.06 (0.78, 1.37)	1.23 (1.07, 1.42)	0.9 (0.59, 1.36)	1.18 (0.91, 1.51)	1.06 (0.78, 1.45)
**NRT_S**	0.89 (0.68, 1.2)	0.95 (0.73, 1.28)	1	1.17 (0.93, 1.52)	0.85 (0.54, 1.38)	1.12 (0.8, 1.59)	1.01 (0.71, 1.5)
**S**	0.76 (0.65, 0.89)	0.81 (0.7, 0.94)	0.86 (0.66, 1.08)	1	0.73 (0.49, 1.08)	0.96 (0.75, 1.21)	0.86 (0.66, 1.14)
**S_ACRT**	1.04 (0.69, 1.55)	1.11 (0.73, 1.69)	1.17 (0.73, 1.84)	1.37 (0.92, 2.03)	1	1.31 (0.82, 2.06)	1.18 (0.74, 1.91)
**S_ACT**	0.79 (0.6, 1.06)	0.85 (0.66, 1.1)	0.9 (0.63, 1.25)	1.05 (0.82, 1.33)	0.76 (0.49, 1.21)	1	0.9 (0.63, 1.31)
**S_ART**	0.88 (0.64, 1.2)	0.94 (0.69, 1.28)	0.99 (0.67, 1.42)	1.16 (0.88, 1.52)	0.85 (0.52, 1.36)	1.11 (0.76, 1.58)	1

S, surgery; NCT_S, neoadjuvant chemotherapy followed by surgery; NRT_S, neoadjuvant radiotherapy followed by surgery; NCRT_S, neoadjuvant chemoradiotherapy followed by surgery; S_ACT, surgery followed by adjuvant chemotherapy; S_ART, surgery followed by adjuvant radiotherapy; S_ACRT, surgery followed by adjuvant chemoradiotherapy.

**Figure 3:**
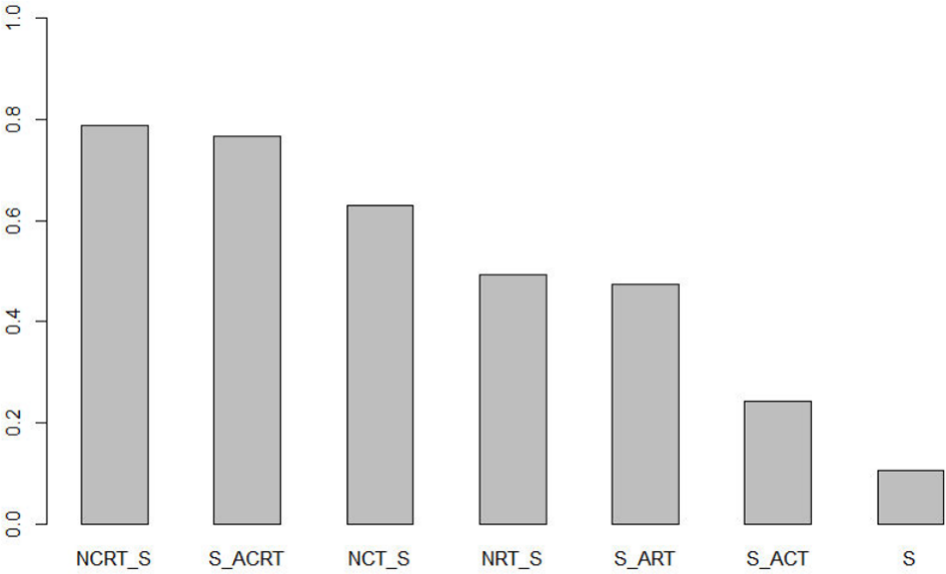
Ranking probabilities.

Ranking analysis (**Figure 3**) was used to provide a hierarchy of treatments. The surface under the cumulative ranking curve value in NCRT was 0.79, which was the highest among all seven treatments, followed by ACRT (0.77), NCT (0.63), NRT (0.50), ART (0.48), ACT (0.25), and surgery alone (0.11). NCRT is most likely to be the optimal treatment modality.

## Discussion

Several previous network meta-analyses (3–5) were conducted but with inconsistent results and some major drawbacks: mixed with adenocarcinoma; including trials with low radiotherapy dose, which may overlook the benefit of radiotherapy adding to surgery; including trials with a small sample size that may not be well randomized; dating back to the 1980s, the time interval between trials was significant, which would enhance heterogeneity; outdated since several essential trials have been published in recent years (6–9). Therefore, we performed a rigorous and updated network meta-analysis including 19 RCTs.

We found that NCRT and NCT showed significantly better survival than surgery alone, while NRT and adjuvant treatments failed to show a survival benefit. Ranking analysis showed that NCRT may be the optimal treatment. This conclusion further strengthens the role of NCRT as the preferred treatment modality in resectable ESCC.

As for neoadjuvant treatments, we found that NCRT and NCT presented a survival benefit compared with surgery alone. Neoadjuvant treatments may have several advantages: tumor downstaging makes the surgical procedure more accessible and improves the radical resection rate; patients could complete the treatment plan before surgery more easily; chemotherapeutic drugs can reach the target before the local blood supply is destroyed. NRT failed to show a survival benefit compared with surgery alone. As a local treatment, radiotherapy plus surgery may exacerbate local toxicity and could not control distant metastasis.

Although NCRT ranked first in the ranking analysis, NCRT did not show a significant survival advantage compared with NCT. Several previous meta-analyses and clinical trials showed the efficacy of NCRT but not NCT. The NeoRes trial (9) revealed slightly improved survival with NCRT among patients with SCC. A meta-analysis (31) showed that NCRT had a significant survival advantage (HR 0.72; 95% CI 0.52–0.99) over NCT in SCC. Several previous meta-analyses and our study showed OS benefit for NCRT is slightly higher than NCT, but the significance for NCRT was not confirmed. Network meta-analyses are generally associated with broader confidence intervals than pairwise comparisons, partly explaining the lack of significance. Nevertheless, these results can only serve as a hypothesis-generating source. It is still not clear whether ESCC will benefit from adding radiotherapy into the neoadjuvant treatment.

Combining chemotherapy and radiotherapy in neoadjuvant therapy may have several advantages: chemotherapy can control distant metastatic tumor cells, and radiotherapy can control regional tumors; therefore, spatial cooperation may enhance antitumor efficacy. Nonetheless, NCRT was also associated with a higher risk of postoperative mortality than neoadjuvant CT or surgery alone (32). The National Comprehensive Cancer Network (NCCN) guidelines recommend NCRT for patients with localized esophageal cancer. In contrast, Guidelines in Japan recommended neoadjuvant chemotherapy for resectable stage II or III thoracic esophageal carcinoma, and for local residual tumor or local recurrence, postoperative irradiation was suggested (33). Therefore, we should balance the benefit and toxicity risks to make individualized treatment choices. Further study is needed to compare survival and toxicity differences between NCRT and NCT to identify whether radiotherapy is indispensable in neoadjuvant treatment.

Adjuvant therapy failed to show a survival benefit compared with surgery alone, though ACRT showed a marginally significant survival advantage and ranked second in the ranking analysis. Adjuvant therapy may eliminate the residual tumor, lymph node metastasis, and possible micrometastasis. However, no visible target could be used to evaluate the effect of postoperative adjuvant therapy, and compliance is poor. Thus, ACRT could be reserved for patients who did not receive neoadjuvant therapy.

There are some limits to our study. First, some of the selected studies contained AC; survival data were extracted from the subgroup analysis for these studies. Despite the random allocation of the studies, the SCC subgroup’s randomization may not be well guaranteed. Second, our report contained trials in a considerable time interval. With the development of surgery techniques, radiotherapy technology, and chemotherapy regimens, heterogeneity between studies cannot be avoided despite careful consideration. Third, our analysis contained only one NRT study. The limited number of patients included should be considered, so the result of NRT compared with other treatment modalities should be interpreted with caution. Last, this study used aggregated data rather than individual patient data; thus, unknown confounding factors may exist.

## Conclusions

For resectable esophageal squamous cell carcinoma, NCRT may be the optimal strategy, and NCT may be the second choice, while other multimodality treatments could not improve OS compared with surgery alone. It remains unclear whether ESCC will benefit from adding radiotherapy into the neoadjuvant treatment.

## Data Availability Statement

The original contributions presented in the study are included in the article/**Supplementary Material**. Further inquiries can be directed to the corresponding author.

## Author Contributions

ZM, MY, YB, and ZH contributed to the conception and design of the study. ZM, MY, and YB organized the database. YW performed the statistical analysis. ZM wrote the first draft of the manuscript. ZM and YW wrote sections of the manuscript. All authors contributed to manuscript revision and approved the submitted version.

## Funding

This study is supported by the National Key Research and Development Program (2017YFC1311000 and 1311002) and CAMS Innovation Fund for Medical Sciences (No. 2016-I2M-1-011).

## Conflict of Interest

The authors declare that the research was conducted in the absence of any commercial or financial relationships that could be construed as a potential conflict of interest.

## Publisher’s Note

All claims expressed in this article are solely those of the authors and do not necessarily represent those of their affiliated organizations, or those of the publisher, the editors and the reviewers. Any product that may be evaluated in this article, or claim that may be made by its manufacturer, is not guaranteed or endorsed by the publisher.
