# Supplementary material for: Survival of Neoadjuvant and Adjuvant Therapy Compared With Surgery Alone for Resectable Esophageal Squamous Cell Carcinoma: A Systemic Review and Network Meta-Analysis
Source: Front Oncol. 2021 Oct 20;11:728185. doi: 10.3389/fonc.2021.728185 (PMC8564474; doi:10.3389/fonc.2021.728185)

Supplement 1. Searching strategy.

(perioperative chemotherapy OR perioperative therapy OR neoadjuvant therapy OR neoadjuvant treatment OR neoadjuvant chemotherapy OR neoadjuvant radiotherapy OR neoadjuvant chemoradiotherapy OR preoperative radiotherapy OR preoperative chemotherapy OR preoperative chemoradiotherapy OR preoperative treatment OR preoperative therapy OR adjuvant therapy OR adjuvant chemotherapy OR adjuvant chemoradiotherapy OR adjuvant radiotherapy OR postoperative care OR postoperative chemotherapy OR postoperative radiotherapy OR postoperative radiation therapy OR postoperative chemoradiotherapy) AND (esophagus cancer OR esophagus carcinoma OR esophagus tumor OR esophagus neoplasms OR esophageal cancer OR esophageal carcinoma OR esophageal tumor OR esophageal neoplasm OR esophageal neoplasms)

Supplement 2. Risk of bias of included studies calculated according to the Cochrane Risk of Bias tool.


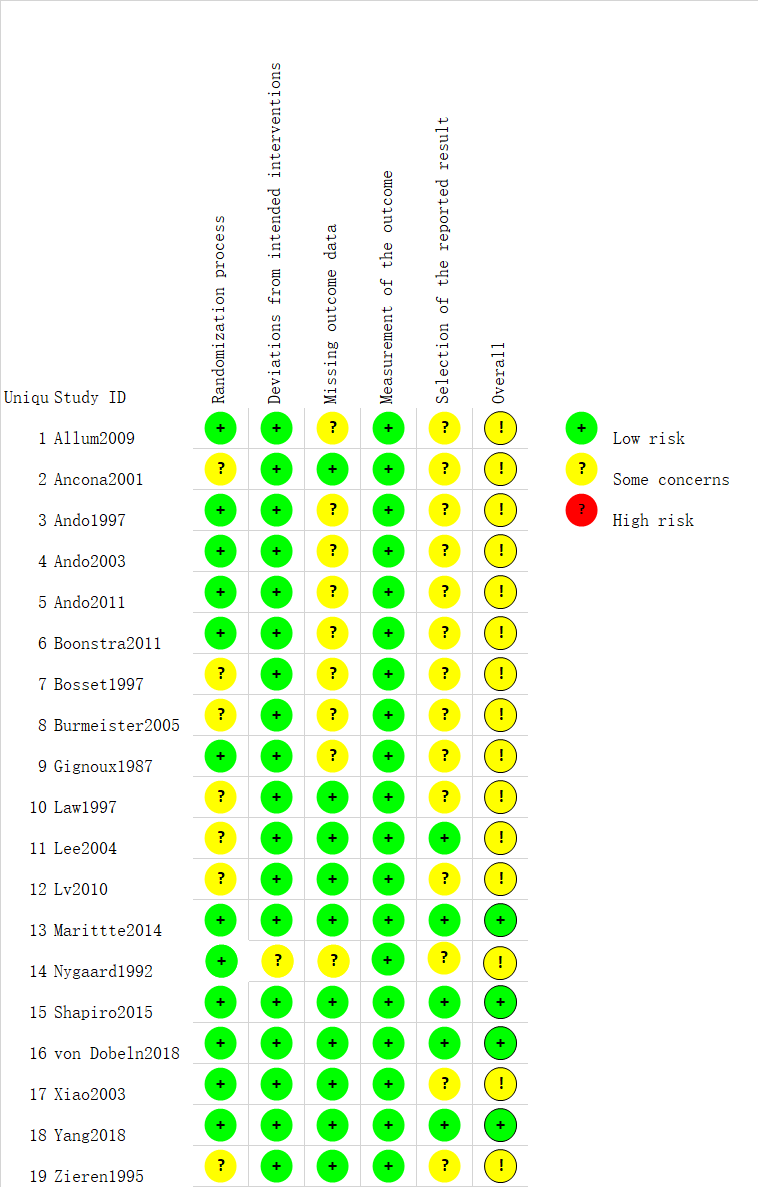


Supplement 3. Node-split models to investigate inconsistency between direct and indirect evidence.


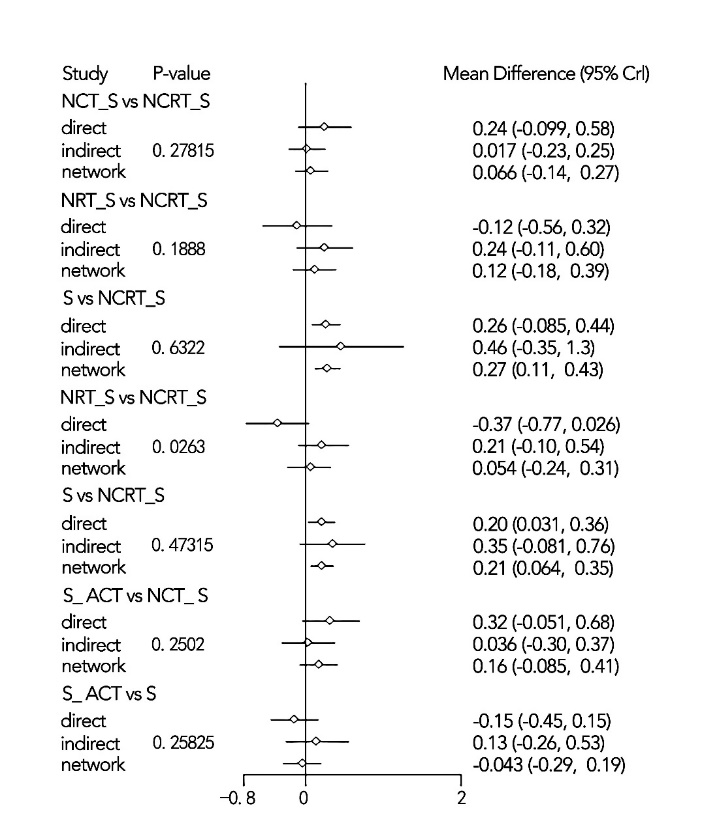


Supplement 4. Funnel plot for risk of publication bias in network meta-analysis.


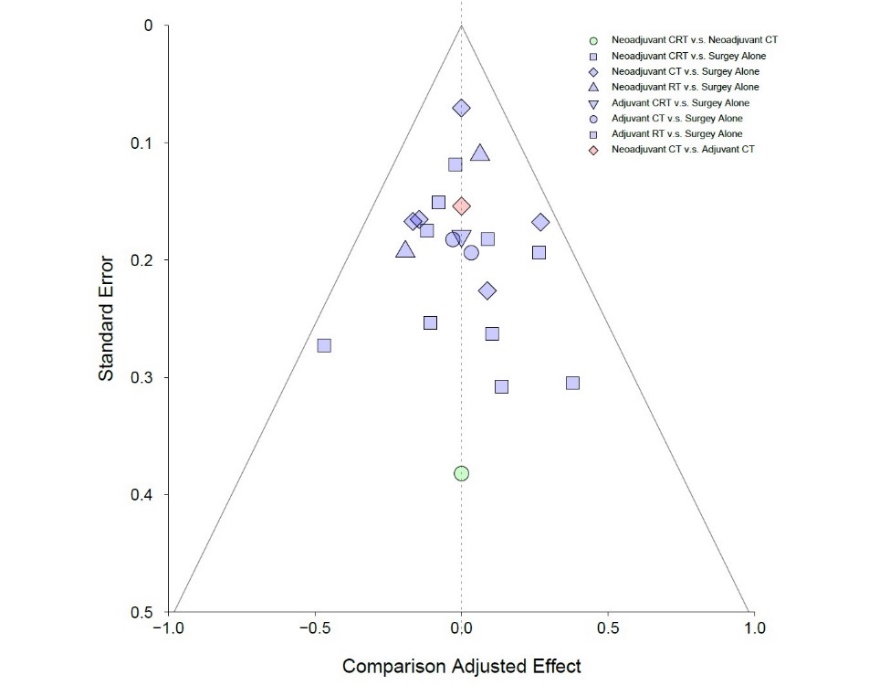

Supplement: Supplementary file 1 [file DataSheet_1.docx]
